# Impact of secondary hematologic malignancies on prognosis of Hodgkin’s lymphoma survivors

**DOI:** 10.3389/fonc.2025.1566063

**Published:** 2025-05-08

**Authors:** Fangheng Lin, Debin Liu, Tao Huang, Yunlong Luo

**Affiliations:** Department of Hematology, Quanzhou First Hospital Affiliated to Fujian Medical University, Quanzhou, Fujian, China

**Keywords:** HL survivors, SHM, prognostic differences, landmark curve, survival analysis

## Abstract

**Background:**

The purpose of this study is to report the differences in the prognosis of Hodgkin’s lymphoma (HL) survivors with or without secondary hematologic malignancies (SHM).

**Methods:**

This study included patients diagnosed with HL in the Surveillance, Epidemiology, and End Results (SEER) database. Propensity score matching was used to balance the baseline differences between SHM and non-SHM patient groups, while survival analysis was used to compare the overall survival and long-term prognosis differences between the two groups.

**Results:**

A total of 36497 patients, along with 231 matched pairs, were included in the study, with a median follow-up time of eight years. The pre-matching multivariate Cox regression results showed that the non-SHM group had a 69% higher risk of all-cause mortality compared to the SHM group. The pre-matching Landmark method revealed no difference in survival between the two groups at < 30 months; at ≥ 30 months, the mortality risk in the SHM group was higher than that in the non-SHM group (HR = 5.188, 95% CI: 3.510, 7.667, P < 0.05). After matching, the Landmark method showed that at < 50 months, the mortality risk of the SHM group was lower than that of the non-SHM group (HR = 0.629, 95% CI: 0.434, 0.935, P<0.05). At ≥ 50 months, the mortality risk of the SHM group was higher than that of the non-SHM group (HR = 3.759, 95% CI: 2.667, 5.300, P < 0.05).

**Conclusion:**

The presence of SHM significantly increases the long-term mortality risk in Hodgkin’s lymphoma survivors.

## 1 Introduction

Hodgkin’s lymphoma (HL) is a lymphocytic malignancy, with an incidence rate of 3-4/100000 per year. It can be diagnosed at any age, and is most common in young and middle-aged individuals (1). In recent decades, significant advancements in HL treatment have greatly improved survival outcomes, establishing HL as one of the most curable cancers (2, 3). Combination therapies involving multiple chemotherapy agents have successfully increased the five-year and ten-year overall survival (OS) of HL to 86% and 80%, respectively. However, prolonged survival also leads to an increased risk of secondary malignancies, cardiovascular disease, or other late effects following treatment (4–7). Further follow-up in cancer databases across North America and Europe indicates that approximately 6.6% to 12% of HL survivors eventually develop second primary malignants (SPMs) (8–10). Especially among survivors of HL in children, the risk of developing secondary malignancies has increased by nearly 20-fold (11). According to the statistical results from the SEER database of the National Cancer Institute (USA), the incidence rate of SPMs in cancer patients is 14.0% higher than that in the general population (12). Andre et al. (13) reported a summary analysis of four randomized trials comparing the long-term OS and toxicity of different chemotherapy regimens for advanced HL, noting that 61 patients developed a total of 63 cases of SPMs (with one patient developing three cases of secondary cancers). There are significant differences in the pathogenesis, clinical manifestations, and treatment approaches between hematologic malignancies and solid tumors. The occurrence of combined secondary hematologic malignancies (SHM) may have unique and complex implications for the prognosis of HL patients, such as increased treatment difficulty and heightened risk of complications. However, the impact of the combined SHM on the prognosis of HL patients remains unclear.

Therefore, based on the SEER (Surveillance, Epidemiology, and End Results) database, this study aims to elucidate the impact of the combined SHM on the prognosis of HL patients, provide robust evidence for the prevention and treatment of HL complicated by SHM, optimize individual treatment strategies, develop more precise and effective treatment approaches, enhance patient management plans, improve long-term prognosis of patients, and elevate their quality of life.

## 2 Method

### 2.1 Data source and population

This study utilized the SEER*Stat program version 8.3.6 (available at https://seer.cancer.gov/seerstat) to extract clinical and pathological information (age, gender, diagnosis year, race, pathological type, primary lesion, Ann Arbor staging, chemotherapy and radiotherapy) along with prognostic data for patients diagnosed with HL from the SEER database between 2000 and 2020. The inclusion criteria were as follows: 1. Patients diagnosed with HL; 2. Patients with secondary hematologic tumors. Exclusion criteria included: 1. Pathological type confirmed by autopsy as cancer, non-HL; 2. Participants diagnosed with hematologic malignancies within two months after HL diagnosis; 3. Missing primary indicators.

### 2.2 Key exposure and outcome indicators

The patients were categorized based on the presence or absence of SHMs. The primary endpoint of this study was OS, defined as the time from diagnosis of HL to death, loss of follow-up, or December 31, 2020.

### 2.3 Definition of SHM and follow-up

SHM was defined as any type of SHM that occurred after HL diagnosis. Curtis and Ries utilized data from Surveillance, Epidemiology, and End Results (SEER) in a 2006 report (14). This database took into account factors such as invasion of the basement membrane by cellular tumors (tumor behavior), histology, origin site, laterality of paired organs (parotid gland and examination), as well as the time since the initial diagnosis to identify multiple primary cancers. Generally, SEER categorizes all metachronous cancers (which develop two months or more after the initial diagnosis) as separate primary cancers unless there is medical record evidence indicating recurrence or metastasis.

The follow-up of SHM began after the diagnosis of HL and concluded on the day of all-cause death or after 21 years of follow-up, whichever occurred first. The deadline for follow-up was defined as December 31, 2020 (SEER data release in November 2022).

### 2.4 Statistical method

Enumeration data were expressed as rates, and comparison between groups was conducted using the chi-square test and Fisher’s exact test. The propensity score matching was employed to match clinical and pathological variables between two groups, thereby minimizing or even eliminating the influence of confounding factors on the study. The matching ratio was set at 1:1, with a caliper value of 0.05. The nearest method was utilized for proportional matching of baseline characteristics between the two groups. Kaplan-Meier (K-M) was used to plot survival curves, and when the hypothesis of equal proportions was satisfied, a log-rank test was applied to assess inter-group differences. Cox proportional hazards regression was used to evaluate the association between the combined SHM and the prognosis of HL survivors; when the curves intersected, the intersection point was identified as a breakpoint for landmark analysis, allowing for the calculation of risk ratios before and after the breakpoint separately. The covariates included in the COX model were age, gender, year of diagnosis, race, pathological type, primary lesion, Ann Arbor staging, chemotherapy and radiotherapy. All statistical analyses were conducted using R software version 4.4.1 (R Project for Statistical Computing), and the difference was considered statistically significant when the p value < 0.05.

## 3 Result

### 3.1 Baseline characteristics of patients before and after matching

A total of 36497 patients were included in this study, with a median follow-up time of 18 years. Patients under 20 years old account for 13.62%, patients aged 20–45 account for 49.97%, patients aged 45–70 account for 26.36%, and patients aged 70 or above account for 0.04%. Among them, 231 patients were in the SHM group and 36266 patients were in the non-SHM group. Before matching, there were differences in basic characteristics between groups. Patients with SHM were more likely to be aged between 45-70, male, diagnosed between 2005 and 2009, and had pathological types of cHL and early stage of Ann Arbor, receive chemotherapy, not receive radiotherapy or provide no information on radiotherapy.After PSM matching, a total of 231 pairs of patients were included in the study. There was no statistically significant difference in clinical record characteristics between the SHM group and the non-SHM group after matching (P > 0.05) (**Table 1**).

**Table 1:** Comparison of baseline characteristics between two groups of HL patients.

Variable		Before PSM			After PSM			
	Total (n = 36497)	No (n = 36266)	Yes (n = 231)	*P*	Total (n = 462)	No (n = 231)	Yes (n = 231)	*P*
Age, n (%)				**<.001^a^**				0.998
<20	4972 (13.62)	4956 (13.67)	16 (6.93)		32 (6.93)	16 (6.93)	16 (6.93)	
≥70	3665 (10.04)	3634 (10.02)	31 (13.42)		62 (13.42)	31 (13.42)	31 (13.42)	
20-45	18239 (49.97)	18158 (50.07)	81 (35.06)		164 (35.5)	83 (35.93)	81 (35.06)	
45-70	9621 (26.36)	9518 (26.24)	103 (44.59)		204 (44.16)	101 (43.72)	103 (44.59)	
Sex, n (%)				**0.011^a^**				1
Female	16522 (45.27)	16437 (45.32)	85 (36.80)		170 (36.8)	85 (36.80)	85 (36.80)	
Male	19975 (54.73)	19829 (54.68)	146 (63.20)		292 (63.2)	146 (63.20)	146 (63.20)	
Year, n (%)				**<.001^a^**				0.999
2000-2004	9368 (25.67)	9276 (25.58)	92 (39.83)		184 (39.83)	92 (39.83)	92 (39.83)	
2005-2009	10014 (27.44)	9941 (27.41)	73 (31.60)		147 (31.82)	74 (32.03)	73 (31.60)	
2010-2014	8664 (23.74)	8621 (23.77)	43 (18.61)		85 (18.4)	42 (18.18)	43 (18.61)	
2015-2020	8451 (23.16)	8428 (23.24)	23 (9.96)		46 (9.96)	23 (9.96)	23 (9.96)	
Race, n (%)				0.315				1
other	6412 (17.57)	6379 (17.59)	33 (14.29)		66 (14.29)	33 (14.29)	33 (14.29)	
unknown	419 (1.15)	418 (1.15)	1 (0.43)		1 (0.22)	0 (0.00)	1 (0.43)	
white	29666 (81.28)	29469 (81.26)	197 (85.28)		395 (85.5)	198 (85.71)	197 (85.28)	
Primary Site, n (%)				0.257				1
lymph nodes	35579 (97.48)	35357 (97.49)	222 (96.10)		444 (96.1)	222 (96.10)	222 (96.10)	
other	918 (2.52)	909 (2.51)	9 (3.90)		18 (3.9)	9 (3.90)	9 (3.90)	
Pathology, n (%)				**0.004^a^**				1
cHL	34493 (94.51)	34285 (94.54)	208 (90.04)		416 (90.04)	208 (90.04)	208 (90.04)	
NLPHL	2004 (5.49)	1981 (5.46)	23 (9.96)		46 (9.96)	23 (9.96)	23 (9.96)	
Stage, n (%)				**<.001^a^**				0.995
early	16812 (46.06)	16709 (46.07)	103 (44.59)		205 (44.37)	102 (44.16)	103 (44.59)	
later	11298 (30.96)	11198 (30.88)	100 (43.29)		201 (43.51)	101 (43.72)	100 (43.29)	
unknown	8387 (22.98)	8359 (23.05)	28 (12.12)		56 (12.12)	28 (12.12)	28 (12.12)	
Chemotherapy, n (%)				0.4				1
No/Unknown	6208 (17.01)	6174 (17.02)	34 (14.72)		67 (14.5)	33 (14.29)	34 (14.72)	
Yes	30289 (82.99)	30092 (82.98)	197 (85.28)		395 (85.5)	198 (85.71)	197 (85.28)	
Radiotherapy, n (%)				0.897				1
None/Unknown	25215 (69.09)	25054 (69.08)	161 (69.70)		322 (69.7)	161 (69.70)	161 (69.70)	
Yes	11282 (30.91)	11212 (30.92)	70 (30.30)		140 (30.3)	70 (30.30)	70 (30.30)	

cHL, classical Hodgkin Lymphoma; NLPHL, Nodular lymphocyte predominant Hodgkin lymphoma. ^a^Significant difference was observed (P < 0.05).

### 3.2 Survival analysis before matching

**Figure 1:**
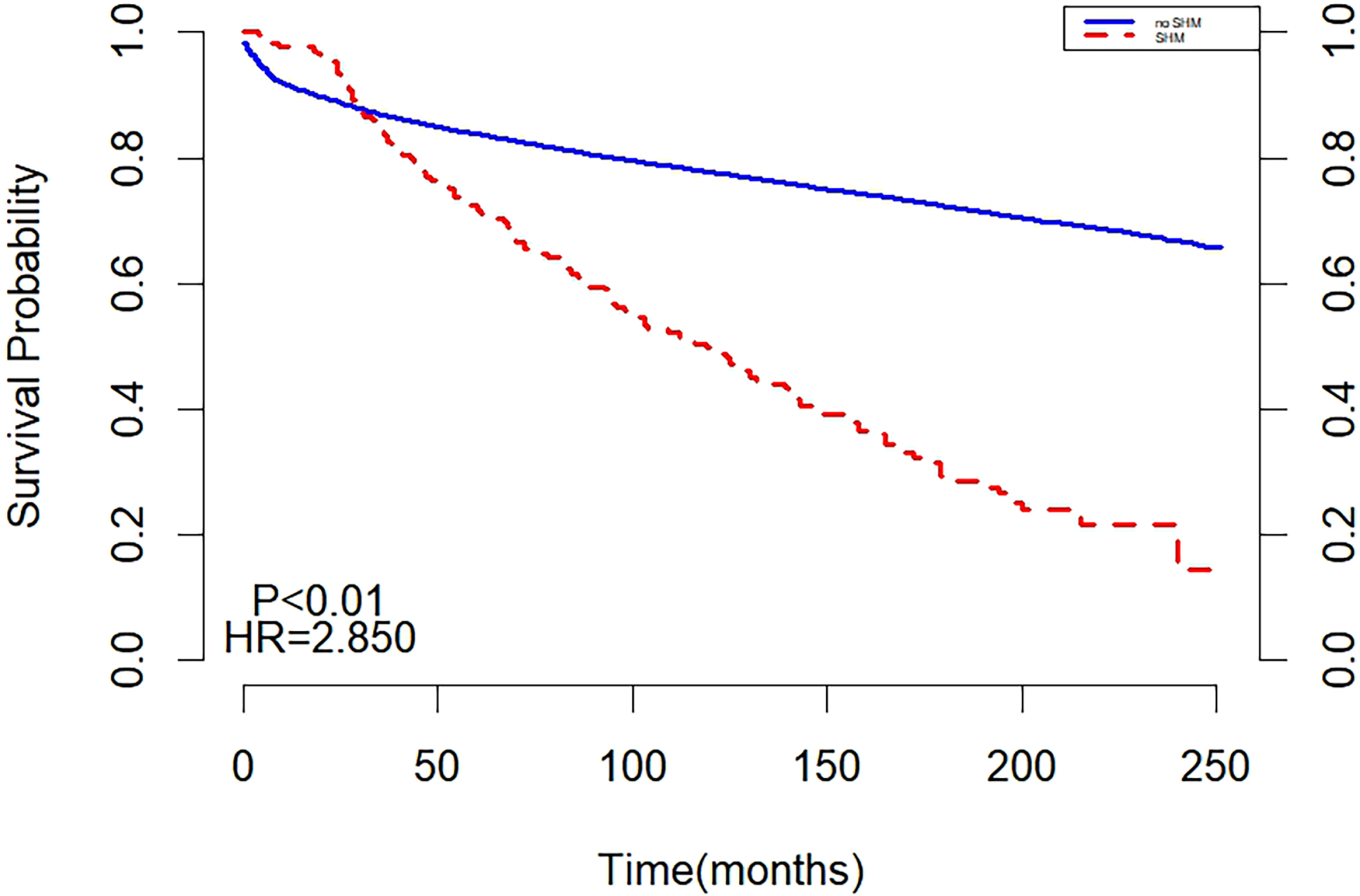
Survival analysis before matching.

Before matching, the two curves in the K-M curve plot represented the survival probabilities of the SHM and non-SHM groups over time. The decline in the SHPM was steeper, with a larger slope compared to the non-SHPM. The curves intersected at 30 months, with the SHM group exhibiting a higher but rapidly decreasing survival probability before the intersection, while the non-SHM group demonstrated a higher survival probability thereafter. The intersection on the K-M curve indicated that the survival of SHM or non-SHM populations did not satisfy the PH assumption, suggesting that the underlying risk varied during different periods of follow-up and necessitating separate analyses for these periods (**Figure 1**).

The results of multivariate cox regression showed that after adjusting for age, gender, year of diagnosis, race, pathological type, primary lesion, Ann Arbor staging, chemotherapy, and radiotherapy, SHM emerged as an independent risk factor affecting the prognosis of HL patients. Throughout the follow-up period, the all-cause mortality risk in the SHM group was 69% higher than that in the non-SHM group (**Table 2**).

**Table 2:** COX table before matching.

Variable	coef	exp(coef)	se(coef)	z	p value^a^	exp(coef)	exp(-coef)	lower.95	upper.95
Age ≥70	3.12	22.71	0.06	49.74	<0.01^a^	22.71	0.04	20.08	25.68
Age 20-45	0.55	1.73	0.06	8.77	<0.01^a^	1.73	0.58	1.53	1.96
Age 45-70	1.81	6.1	0.06	29.4	<0.01^a^	6.1	0.16	5.4	6.88
Sex Male	0.27	1.31	0.02	11.88	<0.01^a^	1.31	0.76	1.25	1.37
Year 2005-2009	-0.15	0.86	0.03	-5.54	<0.01^a^	0.86	1.16	0.81	0.91
Year 2010-2014	-0.32	0.73	0.03	-9.93	<0.01^a^	0.73	1.38	0.68	0.77
Year 2015-2020	-0.21	0.81	0.05	-4.36	<0.01^a^	0.81	1.24	0.73	0.89
Race unknown	-1.65	0.19	0.25	-6.55	<0.01^a^	0.19	5.2	0.12	0.32
Race white	-0.27	0.77	0.03	-9.09	<0.01^a^	0.77	1.31	0.72	0.81
Primary site other	0.18	1.19	0.06	2.95	0^a^	1.19	0.84	1.06	1.34
Pathology NLPHL	-0.75	0.47	0.07	-11.5	<0.01^a^	0.47	2.12	0.42	0.54
Stage later	0.53	1.69	0.03	20.48	<0.01	1.69	0.59	1.61	1.78
Stage unknown	0.01	1.01	0.04	0.28	0.78^a^	1.01	0.99	0.93	1.1
SHPM Yes	0.53	1.69	0.08	6.44	<0.01^a^	1.69	0.59	1.44	1.99
Chemotherapy Yes	-0.56	0.57	0.03	-21.22	<0.01^a^	0.57	1.75	0.54	0.6
Radiotherapy Yes	-0.49	0.61	0.03	-16.3	<0.01	0.61	1.63	0.58	0.65

^a^ Significant difference was observed (P < 0.05).

**Figure 2:**
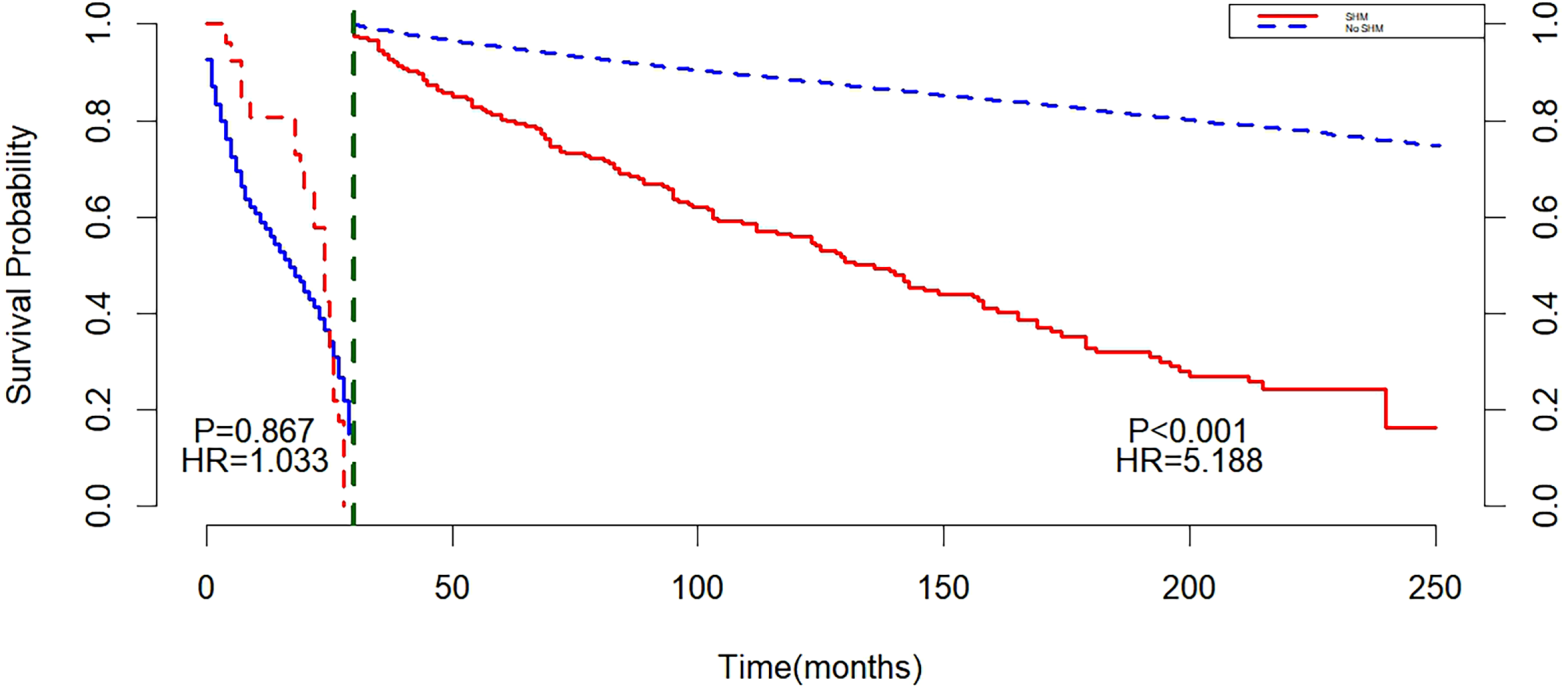
Landmark before matching.

Landmark analysis showed that with 30 months as the cut-off point, there was no statistically significant difference between the non-SHM group and the SHM group before 30 months (P = 0.867). After 30 months, a difference in survival was observed between the SHM group and the non-SHM group (P < 0.001), and the mortality risk in the SHM group was higher than that in the non-SHM group (HR = 5.188, 95% CI: 3.510, 7.667, P < 0.001) (**Figure 2**).

**Figure 3:**
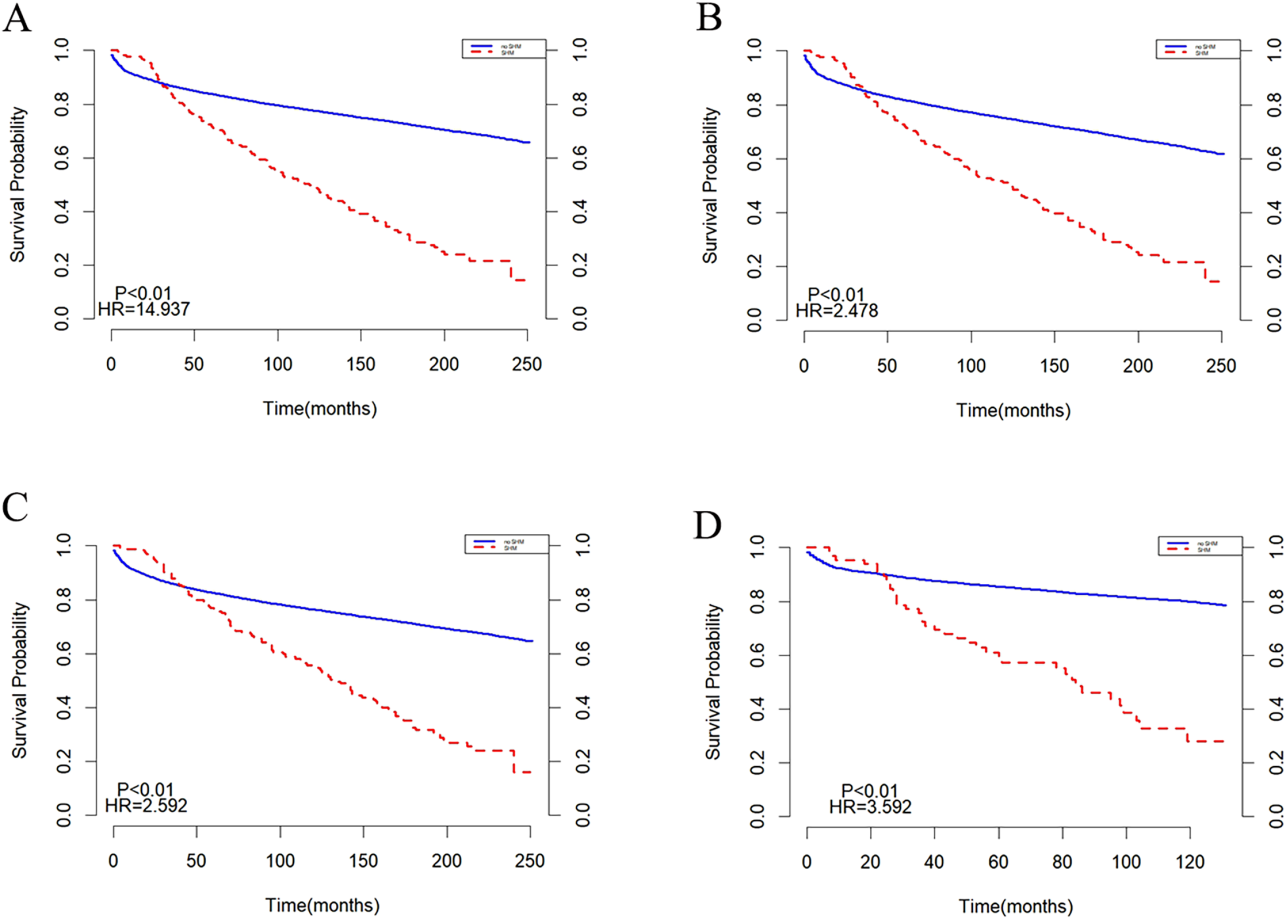
Subgroup analysis before matching: **(A)** age<20, **(B)** age>=20, **(C)** 2000-2009, **(D)** 2010-2020.

Subgroup analyses were conducted based on age and year of diagnosis, with the results presented in (**Figure 3**). In populations aged <20 years and ≥20 years, the survival trends for both the SHM group and the non-SHM group were consistent with those observed in the overall population (**Figures 3A, B**). Similarly, in groups diagnosed between 2000 and 2009, as well as those diagnosed between 2010 and 2020, the survival patterns for the SHM and non-SHM groups mirrored those of the entire population. Notably, within the 2010 to 2020 cohort, the intersection point of the survival curves for the SHM group and the non-SHM group occurred at 20 months, which is earlier than the intersection observed in the overall population (**Figures 3C, D**).

### 3.3 Survival analysis after matching

**Figure 4:**
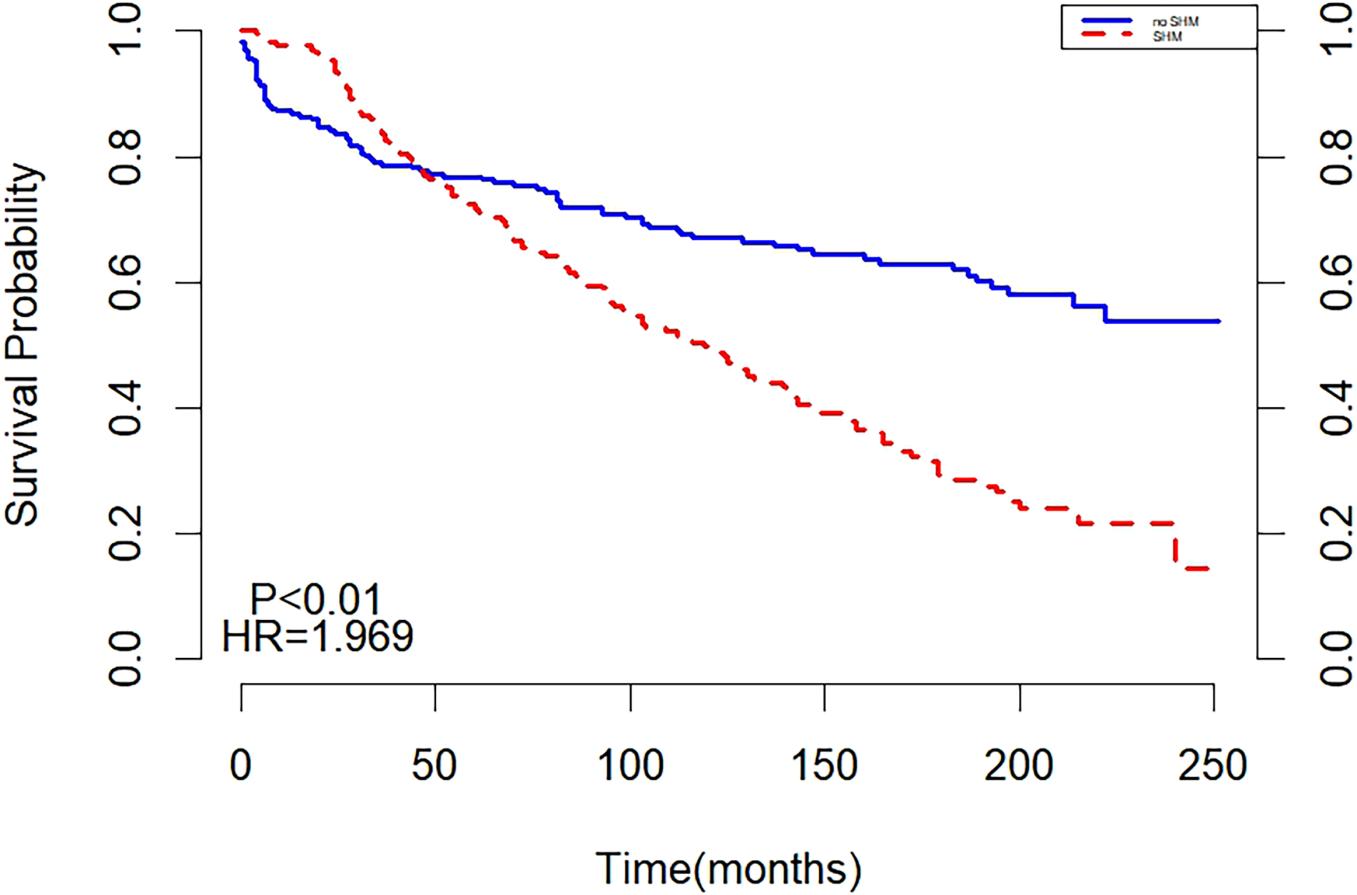
Survival analysis after matching.

After matching, the two curves in the K-M curve plot represented the survival probabilities of the SHM and non-SHM groups over time. The green line showed a significant decline, with a steeper slope compared to the red line. The two curves intersected at 50 months, with the SHM group exhibiting a higher but rapidly decreasing survival probability before the intersection, while the non-SHM group had a higher survival probability afterward. The intersections on the K-M curve indicated that the survival of SHM or non-SHM populations did not meet the PH assumption. This finding suggests that the underlying risk varied during different periods of follow-up, necessitating separate analyses for these intervals (**Figure 4**).

**Figure 5:**
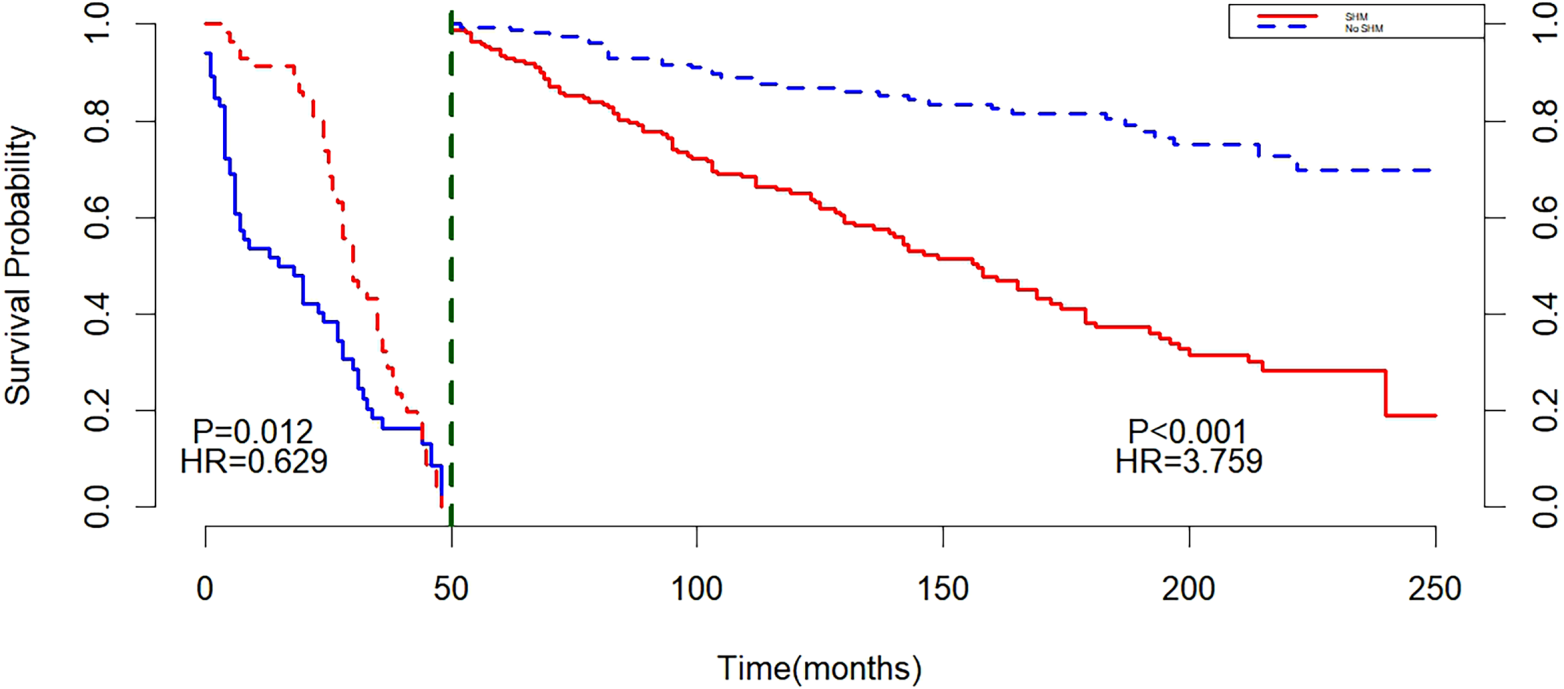
Landmark after matching.

Landmark analysis showed that with 50 months as the cut-off point, a difference in survival was found between SHM and non-SHM groups before 50 months (P = 0.012), and the non-SHM group had a higher mortality risk than the SHM group (HR = 0.629, 95%CI: 0.424, 0.935, P < 0.05). After 50 months, P < 0.001, indicating a difference in survival between the SHM group and the non-SHM group. The mortality risk in the SHM group was higher than that in the non-SHM group (HR = 3.759, 95% CI: 2.667, 5.300, P < 0.01) (**Figure 5**).

**Figure 6:**
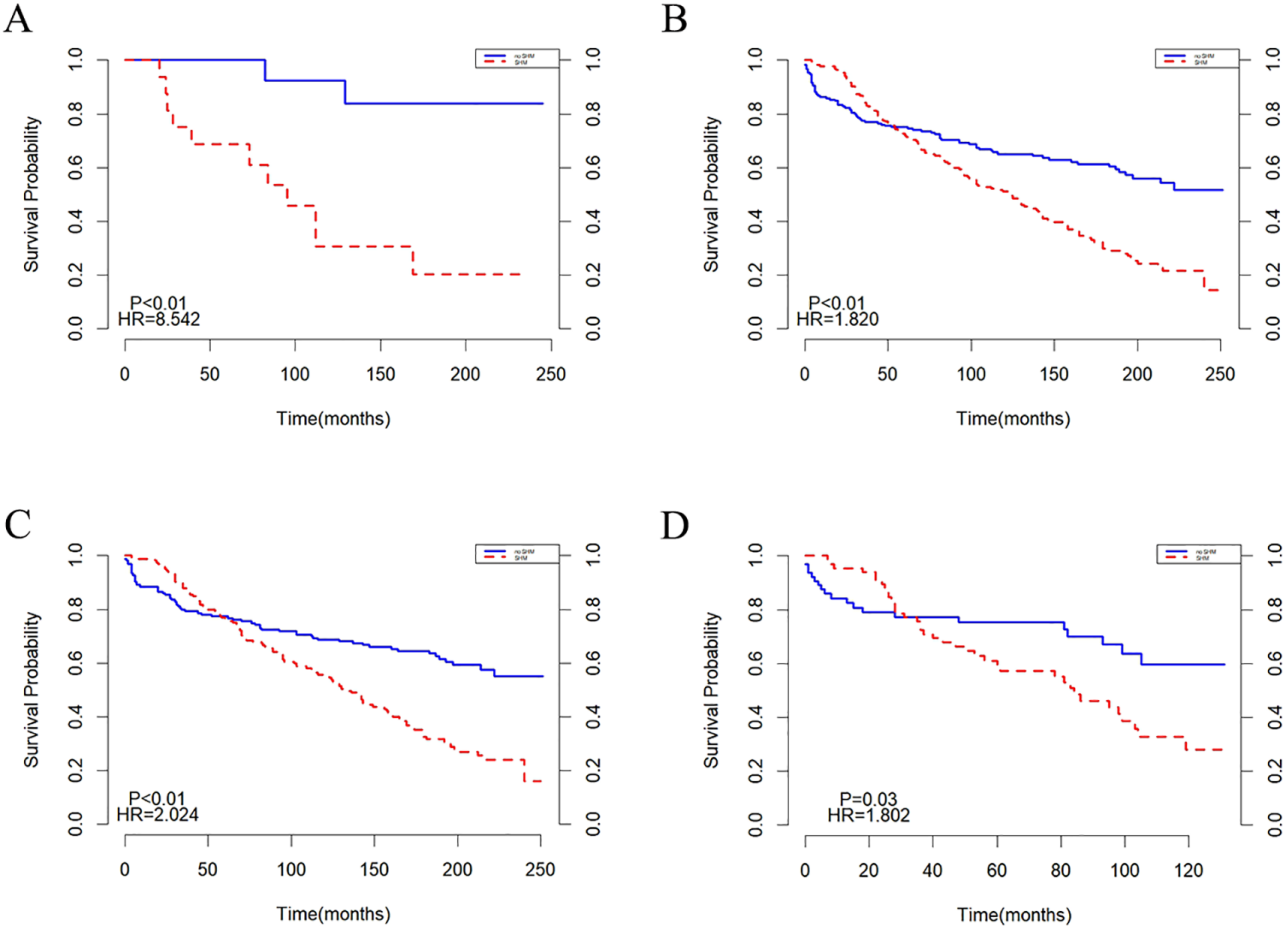
Subgroup analysis after matching: **(A)** age<20, **(B)** age>=20, **(C)** 2000-2009, **(D)** 2010-2020.

Subgroup analyses were performed based on age and year of diagnosis, with the findings illustrated in (**Figure 6**). In the population under 20 years old, the mortality risk for the SHM group was found to be higher than that of the non-SHM group (**Figure 6A**). In contrast, for individuals aged 20 years and older, the survival trends for both the SHM and non-SHM groups were consistent with those of the overall population (**Figures 6A, B**). Similarly, survival patterns in the cohorts diagnosed between 2000–2009 and 2010–2020 also aligned with those of the entire population. However, for the population diagnosed between 2010 and 2020, the intersection point of the survival curves for the SHM and non-SHM groups occurred at 30 months, which is earlier than that observed in the overall population (**Figures 6C, D**).

## 4 Discussion

The incidence rate of the SPMs in patients with hematologic malignancies was 2.04 times that of healthy individuals, with the majority of cases being acute myeloid leukemia/myelodysplastic syndrome (AML/MDS) (12). With the advancement of medical technology, HL patients can achieve long-term survival and a reduced risk of all-cause mortality following cancer treatment (including cytotoxic therapy, radiation therapy, targeted therapy, and immunotherapy). It has become increasingly important to manage the risks associated with secondary cancer and prognostic risks after prolonged survival. Some studies indicate that HL patients are at a heightened risk of developing SPM (5).The study by Dores et al. (15) included 20,007 HL patients (aged 20–74 years) from 2000 to 2015, revealing that second malignancies accounted for 25% of non-HL causes of death. To our knowledge, this was the first large-scale population-based study to demonstrate the prognosis of SHM in HL survivors. We analyzed the survival data of two groups of HL survivors before and after PSM to explore the impact of SHM on their prognosis. The study results reveal that HL survivors face an increased risk of long-term mortality and poorer prognosis due to SHM.

In this study, we found that patients who developed SHM following HL had a significantly elevated risk of long-term mortality. The American Association for Cancer Research (AACR) has released the results from a survival study on the most commonly diagnosed SPM among adult cancer survivors. SPM patients typically exhibit a higher risk of cancer-specific and all-cause mortality compared to first primary malignant (FPM) patients. AML/MDS (acute myeloid leukemia/myelodysplastic syndrome) constitutes a substantial proportion of SHMs. It has been reported that OS and complete response were poorer in secondary AML (s-AML) than in *de novo* AML (16). This condition often arises as a late complication of cytotoxic chemotherapy or radiotherapy used to treat other cancers (such as breast cancer or prostate cancer), or through exposure to environmental carcinogens like benzene. This form of leukemia frequently demonstrates multi-drug resistance during diagnosis, complicating treatment (17). Although the OS of secondary AML has improved slightly over the past few decades, it remains low (18, 19). The only confirmed treatment for s-AML at present is allogeneic hematopoietic cell transplantation. A previous study showed that adolescents and young people with SPM were more likely to experience poorer survival compared to those with the same primary hematologic malignancies (20).These studies all suggested that patients with SPM had a worse prognosis, but the specific reasons were still unclear. A meta-analysis focusing on cancer treatment-related myeloid neoplasm revealed that cancer treatment-selected for clones with mutations in the DNA damage response (DDR) genes TP53, PPM1D, and CHEK2, which were significantly enriched in therapy-related myeloid neoplasm (tMN). In the absence of cytotoxicity or radiation therapy, these clones had lower competitive fitness compared to non-DDR gene mutations. It indicated that in the context of cancer treatment, clones with DDR gene mutations outperformed other clones, thereby promoting tMN (21). Patients with TP53 mutant hematologic malignancy display heightened resistance to cytotoxic drugs that induce cancer cell death through DNA damage, and exhibit deficiencies in metabolism, genomic stability, and autophagy. As a result, they often display lower sensitivity to multiple anticancer drugs (22), contributing to a poor prognosis. HL and its treatments may suppress immune function through chronic inflammation and the inhibition of cellular defense mechanisms, creating opportunities for malignant cells to evade immune surveillance and facilitating viral reactivation, which can subsequently promote the development of SHM (23). For these treatments, radiotherapy can further enhance immune suppression, altering the tumor immune microenvironment. This alteration includes the upregulation of PD-L1, increased expression of CD73, the release of immunosuppressive chemokines, and the activation of TGF-β family members mediated by the ATR/CHK1 pathway, all of which are associated with a poor prognosis in SHM (24). The SHMs included in this study encompassed but were not limited to AML/MDS. The overall results showed that SHM patients experienced a decreased long-term survival and an increased risk of death, which was consistent with previous study reports. However, in this study, through propensity score matching and Landmark analysis, we found that the survival of SHM patients was significantly better than that of non-SHM patients when the follow-up time was less than 50 months. Patients with SHM may receive closer follow-up and more aggressive treatment following a secondary cancer diagnosis, which can help better manage their condition in the short term and yield more comprehensive short-term data. Early interventions may prioritize high-intensity regimens, such as chemotherapy combined with immunotherapy, to rapidly reduce the tumor burden (25). However, good short-term efficacy did not necessarily translate to a favorable long-term prognosis. This study showed that when the follow-up period was extended to 50 months or more, the survival of SHM patients was significantly lower than that of non-SHM patients. Several factors contributed to the poor long-term outcomes associated with SHM. In addition to the complex genetic background of secondary malignants and the increased challenges in diagnosis and treatment due to a decline in the overall health status of patients, it was also closely related to various factors such as patients’ treatment willingness and expectations, long-term treatment resistance and side effects, accessibility of medical resources, and economic and psychological support from families and society. In addition, patients with a history of hematologic malignancies often experience complications such as pathogen colonization, recurrent bleeding episodes, and transfusion dependence when SHM occurs, which significantly elevate mortality rates (26–28). Certainly, during long-term follow-up, some patients may discontinue medical monitoring (such as transferring to primary care hospitals), which could lead to biases by underreporting non-tumor-related mortality events. Due to the lack of cytogenetics, genomic data, and specific chemotherapy drug data in the SEER database, this study did not further analyze potential factors related to poor prognosis.

### 4.1 Variations in the risk of SHM in younger HL survivors

Given the excellent long-term survival rates of HL and its high incidence among adolescents, the emphasis on long-term toxicity often surpasses the focus on improving treatment efficacy. The initial chemotherapy agents used in pediatric regimens are comparable to those used in adult protocols; however, recognizing the increased risk of long-term effects in younger patients has prompted the development of tailored treatment strategies that consider the cumulative doses of alkylating agents, doxorubicin, bleomycin, and radiotherapy (7, 29). This raises the question: will there be differences in the prognosis of SHM among HL survivors due to variations in age, adult versus pediatric treatment approaches, and the years of study? This investigation performed a subgroup analysis, both pre- and post-matching for individuals aged 20, revealing that the risk of death from SHM in children, adults, and the overall HL population remained largely similar. In 2024, Chinese scholars published a study utilizing the SEER database, which encompassed a total of 6,984 cases of adolescent lymphoma diagnosed between 1975 and 2018. This study found that the cumulative incidence rate had not shown signs of deceleration over the past 40 years, but rather was on the rise. Moreover, their findings indicated that chemotherapy alone did not increase the incidence of SHM; however, it notably accelerated the onset of SHM in children with HL, with an occurrence approximately six years earlier than expected (30). Additionally, subgroup analysis based on diagnosis years—comparing 2000–2009 and 2010-2020—revealed that HL patients diagnosed between 2010 and 2020 exhibited a significantly earlier risk for developing SHM. The earlier onset of SHM in HL patients may be tied to several factors. Although modern radiotherapy techniques have reduced dosages and irradiation fields, they may still inflict long-term damage to sensitive tissues in young patients (31), potentially contributing to the earlier development of SHM. A study by Taylor et al. in 2009 investigating the risk of thyroid cancer in childhood cancer survivors highlighted the importance of age at initial treatment in assessing long-term cancer risk. Specifically, younger age at initial treatment correlated with an increased risk of SHM (32). Genetic predisposition may play a significant role in the development of secondary neoplasms. Patients with cancer predisposition syndromes are more susceptible to the late effects of chemoradiotherapy. Genetic polymorphisms in the enzymes that metabolize chemotherapy drugs are also suspected to play a role in the pathogenesis of therapy-related myelodysplastic syndromes/acute myeloid leukemia (tMDS/AML) (33). Furthermore, advancements in imaging and molecular diagnostic technologies have allowed for the earlier detection of SHM, which may contribute to the observation of younger ages at onset. The prolonged survival of HL patients inherently increases the cumulative risk of developing secondary SHMs, especially among younger patients who have more time to develop subsequent tumors (34). In recent years, the introduction of BV and anti-PD-1 antibodies has disrupted the traditional treatment landscape, leading to significant changes in HL management. Consequently, with ongoing long-term follow-up, the risk of SHM in HL patients may continue to evolve.

There is currently no standardized diagnosis and treatment specification for HL patients complicated with SHM, which suggests that strengthening SHM monitoring may be beneficial for patients at high risk of developing SHM. Importantly, the difference in SHM risk is closely related to the type of cancer that survivors have previously suffered from (35), suggesting the need for individualized strategies for managing SHM patients. A population-based cancer registry review in Ontario found that, despite established cancer screening interventions, SPM screening results for lymphoma patients were insufficient (36). To raise awareness of the importance of follow-up among lymphoma survivors and clinical doctors, specialized examination protocols should be implemented. Tailored survival plans should be used to ensure more comprehensive attention to patients and improve compliance for early detection of SHM. In terms of treatment for SHM, while it is crucial to consider the types and stages of new malignants, we should also take into account the primary malignants and physical tolerance. However, future researchers will also strive to develop a screening risk assessment model for SHMs (37, 38) to evaluate the potential risk of developing SHM in the treatment of various types of cancer. Regular follow-up and monitoring of patients are essential, with a focus on screening for secondary tumors and strengthening pharmacovigilance research to address the specific risks associated with various populations and treatment regimens. Studies have identified that HL survivors carrying BRCA1/2 mutations face a significantly heightened risk of developing secondary hematologic malignancies (SHM) and should therefore receive early intervention (39). It is recommended to provide more targeted, stratified, and precise treatment for newly diagnosed SHM patients through NGS or immunohistochemical evaluations (40). Patients who test positive for SHM should be monitored for the presence of Epstein-Barr virus (EBV) DNA, which indicates a risk of recurrence (41). Additionally, lung function testing should be conducted to screen for post-radiotherapy pulmonary fibrosis (42) and thyroid ultrasounds should be performed to assess for anthracycline-related thyroid toxicity (43). During disease progression, it is important to reevaluate genomic factors (44) and monitor for potential clonal evolution resulting from treatment, such as the accumulation of drug-resistant mutations, ensuring timely adjustments to treatment plans. The selection of treatment strategies should take into account both short-term prognoses and long-term survival. Low-toxicity treatment options should be prioritized, and clinical trials involving innovative therapies (such as CAR-T, targeted drugs, and demethylation agents (45)) should be recommended to effectively balance tumor control in HL while enhancing patients’ long-term quality of life and maximizing the survival benefits for SHM patients. It should be a key point to continue to develop new and effective drugs and cancer treatment strategies with fewer complications.

The strength of this study lies in the relatively uniform large observation population sourced from the SEER database, and the use of propensity score matching to minimize potential bias associated with the absence of randomization, leading to relatively reliable conclusions. However, this study does have limitations. Firstly, the lack of randomization in the initial treatment of HL may introduce potential biases. Secondly, the SEER database only captures initial treatment information for HL, making it unclear whether HL patients have received during subsequent treatment. While this limitation is unlikely to affect our main conclusion, it may lead to an underestimation of the increased risk of chemotherapy.

## 5 Conclusion

In conclusion, our large-scale population-based study indicates that the presence of SHM significantly increases the long-term mortality risk in Hodgkin’s lymphoma survivors. Based on this, we should strive to improve doctors’ and patients’ understanding of SHMs, provide effective prevention and follow-up strategies, achieve early detection and treatment, and contribute to improving patients’ prognosis.

## Data Availability

The original contributions presented in the study are included in the article/supplementary material. Further inquiries can be directed to the corresponding author.
